# Supplementary material for: Simultaneous Control of Staphylococcus aureus and Bacillus cereus Using a Hybrid Endolysin LysB4EAD-LysSA11
Source: Antibiotics (Basel). 2020 Dec 14;9(12):906. doi: 10.3390/antibiotics9120906 (PMC7764928; doi:10.3390/antibiotics9120906)
Supplement: Supplementary file 1 [file antibiotics-09-00906-s001.pdf]

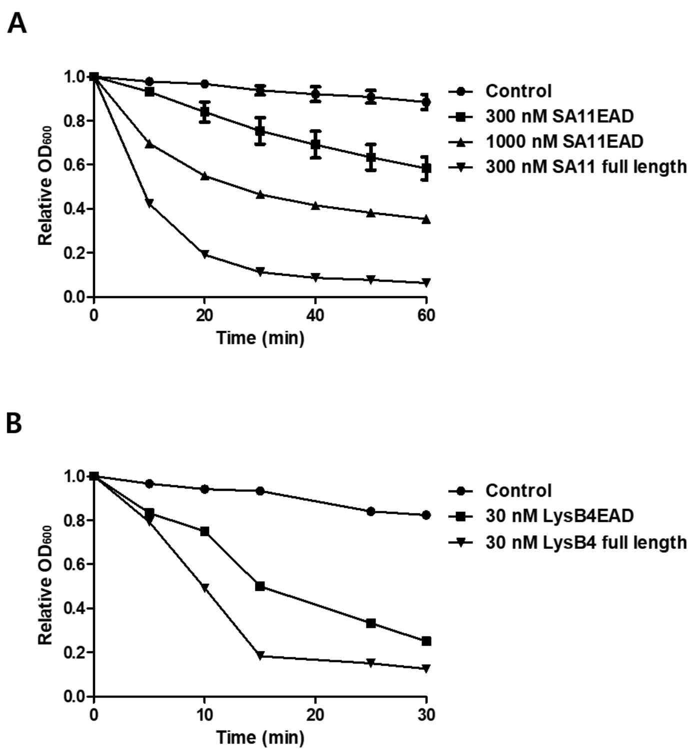

Figure S1. The lytic activity of LysSA11, LysB4 and their EADs. (A) LysSA11 and LysSA11-EAD were added to the suspension of *S. aureus* RN 4220. (B) LysB4 and LysB4-EAD were added to the suspension of *B. cereus* ATCC 21768, and the decrease of turbidity was monitored.

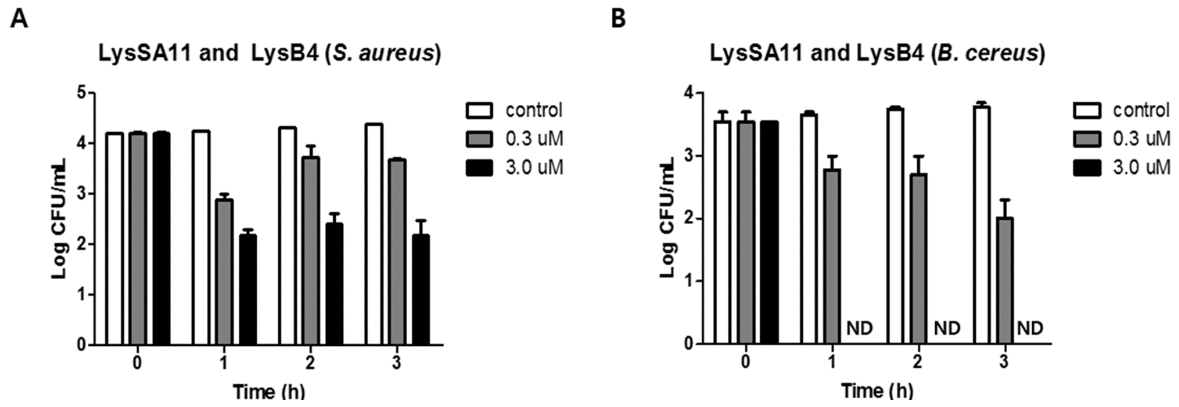

Figure S2. The antimicrobial activity of LysSA11 and LysB4 in combination in the boiled rice contaminated simultaneously with *S. aureus* and *B. cereus*. The numbers of *S. aureus* RN4220 (A) and *B. cereus* ATCC 21768 (B) cells in boiled rice were counted after treatment with different concentrations (0.3 μM and 3.0 μM) of LysSA11 and LysB4 in combination. ND, not detected.

Table S1. The decrease in OD600 values of *S. aureus* after addition of endolysins

Time (min)	Control		LysSA11		LysSA11-LysB4		LysB4-LysSA11		LysSA11-LysB4EAD		LysB4EAD-LysSA11	
0	0.156	0.1455	0.1296	0.1334	0.149	0.1476	0.1505	0.1482	0.1335	0.1383	0.1352	0.14
10	0.1543	0.1426	0.0306	0.044	0.0368	0.0557	0.1463	0.1431	0.0336	0.0342	0.1233	0.1214
20	0.1424	0.1393	0.0304	0.0355	0.0362	0.0365	0.099	0.1305	0.0335	0.0338	0.0953	0.0889
30	0.1314	0.134	0.0301	0.0346	0.0358	0.0362	0.0518	0.0975	0.033	0.0335	0.0716	0.0629
40	0.124	0.1332	0.0297	0.0344	0.0352	0.0362	0.0399	0.0627	0.033	0.0331	0.0587	0.0523
50	0.1235	0.1308	0.0302	0.0342	0.0357	0.0359	0.0381	0.0454	0.0326	0.0327	0.0523	0.0473
60	0.1216	0.1307	0.03	0.0342	0.0354	0.0362	0.038	0.0394	0.0324	0.0328	0.0489	0.0447

Table S2. The decrease in OD600 values of *B. cereus* after addition of endolysins

Time (min)	Control		LysB4		LysSA11-LysB4		LysB4-LysSA11		LysSA11-LysB4EAD		LysB4EAD-LysSA11	
0	0.1577	0.13	0.1191	0.1206	0.1493	0.1247	0.1509	0.1306	0.13	0.1232	0.1306	0.1349
10	0.1476	0.1251	0.0359	0.0417	0.0469	0.0622	0.0363	0.0502	0.0453	0.0455	0.0652	0.0762
20	0.1482	0.1164	0.036	0.0417	0.0361	0.0487	0.0358	0.0494	0.045	0.045	0.061	0.0704
30	0.1178	0.1145	0.0357	0.0417	0.0354	0.0484	0.0354	0.0491	0.0446	0.044	0.0585	0.0659
